# Associations between 24-hour movement behavior profiles and mental health among Chinese school students: A latent profile analysis

**DOI:** 10.1016/j.jesf.2026.200515

**Published:** 2026-08-22

**Authors:** Ting Li, Li-Juan Wang, Chang Zhang, Yu-Wei Liu, Yan-Ping Qiu, Guo Liang

**Affiliations:** School of Physical Education, Shanghai University of Sport, Shanghai, China

**Keywords:** 24-h movement behavior, Pattern, Mental health, Latent profile analysis

## Abstract

**Background:**

This study aimed to identify 24-h movement behavior profiles on weekdays (WDs) and weekends (WEs) among primary and secondary school students in China and investigate the associations of these profiles with mental health.

**Methods:**

The study recruited 400 primary school students (mean age: 9.4 ± 1.9 years; 47.8% girls) and 819 secondary school students (mean age: 14.9 ± 1.4 years; 52.0% girls). Accelerometers were used to measure 24-h movement behavior, including sedentary behavior (SB), light physical activity (LPA), moderate-to-vigorous physical activity (MVPA), and sleep. Mental health included depressive symptoms, internalizing/externalizing problems, working memory, and inhibition control. Latent profile analysis was used to identify 24-h movement behavior profiles.

**Results:**

Three profiles were identified among primary school students: “consistently low SB/high MVPA/long sleep”, “consistently high LPA”, and “consistently high SB/low PA/short sleep”. The “consistently low SB/high MVPA/long sleep” and “consistently high LPA” groups had significantly lower depression levels, fewer internalized problems, better working memory, and better inhibition control than the other profiles. In secondary school students, “low MVPA (WDs) and low MVPA/high LPA (WEs)”, “consistently high SB/low LPA/short sleep”, “low SB/High LPA (WDs) and low SB/long sleep (WEs)”, and “high MVPA/long sleep (WDs) and high MVPA (WEs)” were identified. The “high MVPA/long sleep (WDs) and high MVPA (WEs)” group had significantly lower depression levels and fewer internalized problems than the “consistently high SB/low LPA/short sleep” group.

**Conclusion:**

The study highlights the importance of promoting healthy 24-h movement behavior patterns. Mental health promotion strategies should aim to optimize 24-h movement behaviors (i.e., increasing MVPA, decreasing SB, and ensuring adequate sleep duration) or maintain high levels of LPA for primary school students. For secondary school students, they should focus on prioritizing ensuring adequate sleep and encouraging regular participation in MVPA.

## 1 Background

The mental health conditions of children and adolescents, such as depression disorders and emotional and behavioral problems, have become serious problems in China.^1^^,^^2^ The age-standardized prevalence of mental disorders among children and adolescents in China has increased from 4.8% in 1990 to 8.9% in 2021.^3^

A growing body of research shows that physical activity and adequate sleep may be associated with better mental health through multiple biological and psychological pathways, including enhanced neuroplasticity, regulation of neurotransmitter systems, restoration of circadian rhythms, and improved emotion regulation and stress management.^4^, ^5^, ^6^ Evidence utilizing variable-centered analysis also suggested that the 24-h movement behavior, including moderate-to-vigorous physical activity (MVPA), light physical activity (LPA), sedentary behavior (SB), and sleep, is independently associated with the mental health of children and adolescents.^2^^,^^7^^,^^8^ Furthermore, a recent compositional data meta-analysis including 19 studies showed that engaging in more MVPA, getting more sleep, and spending less time in SB was favorably associated with social–emotional health.^9^ However, variable-centered analyses may overlook heterogeneity in how movement behaviors cluster within individuals, limiting the understanding of how different combinations of movement behaviors influence mental health.^10^ A person-centered approach must be adopted to resolve this problem. This analytical approach categorizes individuals into distinct subgroups based on their multidimensional movement behavior characteristics, thereby capturing the heterogeneity of movement behavior patterns within the population.^11^ Compared with the variable-centered paradigm, this approach provides a more nuanced characterization of subgroup-specific features, uncovers potential differences between behavioral subgroups, and facilitates the development of tailored interventions based on subgroup-specific behavioral characteristics.^12^, ^13^, ^14^ One such person-centered analytical method is latent profile analysis.

To the authors' knowledge, only two previous studies have utilized the latent profile analysis to understand the relationship among the self-reported MVPA, recreation screen time, sleep, and mental health of adolescents.^15^^,^^16^ Brown, Kwan et al.^16^ examined the association between movement behavior guideline adherence and depressive symptoms of adolescents. Four profiles were identified: one healthy profile (high MVPA/low screen time), two mixed behavioral profiles (low MVPA/low screen time and high MVPA/high screen time), and one profile considered to be the least healthy (low MVPA and high screen time). Depressive symptoms in adolescents were lowest among those with the healthy profile, and this trend was evident 1 year later. Brown, Cairney et al.^15^ further investigated the link between these four movement behavior profiles and adolescents’ mental well-being, and they achieved similar findings. Specifically, the healthy profile was consistently linked to enhanced mental well-being, followed by the mixed behavior profiles and the least healthy profiles.

However, these two studies are limited by the use of self-reported measures of movement behavior, the ignorance of the compositional nature of the movement behavior data, and the differences in movement behavior between weekdays (WDs) and weekends (WEs). First, self-reported measures of movement behavior may result in recall bias. Second, movement behavior profiles on WDs and WEs are inherently different due to learning-related tasks. A large body of evidence showed that children and adolescents have delayed sleep, more SB, and less PA on WEs than on WDs.^17^, ^18^, ^19^ However, these two studies did not consider the possible heterogeneity in movement behavior profiles and their association with mental health between WDs, which are characterized by consistent, structured, less autonomous, and segmented days with adult supervision and WEs, which have less structure and occur in a more autonomous environment. Third, the movement behavior across the 24-h day constitutes “compositional” data, which consist of mutually exclusive and exhaustive parts (time spent in MVPA, LPA, SED, and sleep) of a whole (24-h movement).^20^ Analysis of 24-h time use is now widely accepted as an approach that respects the compositional properties of the data. These two studies identified movement behavior profiles without accounting for compositional properties of time-use data, which may lead to a possible misclassification when using latent profile analysis.^20^^,^^21^

In China, studies have been conducted to examine the relationships between 24-h movement behavior and adiposity,^22^^,^^23^ physical fitness,^24^^,^^25^ and mental health.^26^^,^^27^ Moreover, Cao et al.^28^ applied two-step cluster analysis based on self-reported PA, screen time, and sleep time to identify lifestyle patterns among adolescents in northeastern China. They found that, compared with students in the active pattern, those in the high screen time pattern were more likely to experience depressive symptoms. However, this study did not account for the compositional nature of 24-h movement behaviors. Evidence remains limited regarding objectively measured 24-h movement behavior profiles identified using a compositional data approach and their associations with psychological health among China school students. The current study aimed to identify 24-h movement behavior profiles on WDs and WEs among children and adolescents in China by using a compositional latent profile approach and investigate their association with mental health. The World Health Organization defines mental health as a state of mental well-being in which individuals can cope with the stresses of life, realize their abilities, and contribute to their community, encompassing both social–emotional health (e.g., depression, internalizing and externalizing symptoms) and cognitive functioning (e.g., inhibition ability and memory).^9^ Accordingly, these dimensions were included as outcomes in the present study. Notably, primary school students (6–12 years) have different movement behavior patterns from secondary school students (13–18 years, e.g., children have more PA and longer sleep duration than adolescents) based on previous research evidence,^29^, ^30^, ^31^ so the association between 24-h movement behavior patterns and mental health may differ between these age groups. Therefore, all analyses in the present study were conducted separately for primary and secondary school students. The findings could assist in devising targeted and effective interventions for primary and secondary school students’ mental health, respectively.

## 2 Method

### 2.1 Data collection

Data collection was conducted from May to June 2025 by the first author and two research assistants. The purpose of the research was introduced to the participating children and adolescents, and informed consent was obtained from the participants and their parents. Each participant was provided with an accelerometer and instructed to maintain their usual daily routines throughout the monitoring period. Accelerometers were retrieved after 8 days to ensure 7 days of complete data collection. Thereafter, a questionnaire survey, including the mental health assessment, was administered to the participating primary and secondary school students with valid accelerometer data. The self-reported survey was conducted among all participating secondary school students aged 13–18 years during regular school hours. For primary school students aged 6–12 years, the questionnaires and instructions were placed in their “take-home” folder for their parents to complete and returned to the teachers the next day.

### 2.2 Participants

Participants were recruited from two primary schools and four secondary schools in Wuhan and Changsha, the mid-south region of China. Two or three classes were randomly selected from each grade (grades 1–6, 7 and 8, and 10 and 11) in each school. Students in grades 9 and 12 declined to participate in the study due to tight academic learning schedules associated with preparation for upcoming high school and university entrance examinations. A total of 2014 participants were invited to join the study, and 1747 participants agreed to participate. Among them, 528 participants were excluded on the basis of the following exclusion criteria: (1) severe chronic diseases and physical disability (seven participants), (2) accelerometer malfunction or loss (33 participants), (3) valid accelerometer data not covering at least two valid WDs and one valid WE (73.6% efficiency, 461 participants), and (4) lack of mental health indicators and confounder information (27 participants). In total, 400 primary school students and 819 secondary school students participated in the study. Comparisons between included (n = 1219) and excluded participants (n = 528) showed no significant differences in sex (χ^2^ = 0.953, *P* = 0.329), age (t = −1.287, P = 0.198), paternal education level (χ^2^ = 3.629, *P* = 0.459), maternal education level (χ^2^ = 5.585, *P* = 0.232), or household income (χ^2^ = 3.314, *P* = 0.346). The study was approved by the Ethics Committee of Shanghai University of sport (102772023RT090).

### 2.3 Measures

#### 2.3.1 24 h movement behaviors

24 h movement behaviors were measured using Actigraph wGT3X-BT accelerometers (Pensacola, FL, USA). Participants were instructed to wear the accelerometer on the right hip for 7 consecutive days of continuous 24 h, except during any water-based activities. Raw accelerometer data were downloaded in 15 s epochs and analyzed using Actilife software (version 6.13.3). After the total nocturnal sleep was accounted for, a valid day was defined as ≥ 10 h of wear time on days, and participants were included in the analysis if they had a minimum of two valid WDs and one valid WE.^32^^,^^33^ Non-wear time was defined as ≥ 20 consecutive minutes of zero-intensity counts.^34^ Non-sleep time was categorized into SB ≤ 100 counts/min (CPM), LPA (101–2295 CPM), and MVPA (≥2296 CPM), based on the cut-points established by Evenson et al..^35^ Data were subsequently aggregated into 60 s epochs to obtain sleep period time by using a fully automated algorithm for 24-h waist-worn accelerometers, which was recently validated in the International Study of Childhood Obesity, Lifestyle and the Environment.^33^^,^^36^ Briefly, sleep time was the amount between algorithm-determined sleep onset and sleep offset. A night was considered valid if overnight sleep duration was ≥160 min. Participants with at least three valid nights of sleep, including at least one weekend night, were included in the analyses. All sleep data were then visually inspected and confirmed independently by two researchers.

#### 2.3.2 Mental health

##### 2.3.2.1 Depressive symptoms

Depressive symptoms were assessed using the Center for Epidemiologic Studies Depression Scale Revised.^37^ This scale comprises 20 items that measure symptoms of depression across nine categories (sadness, anhedonia, appetite, sleep, thinking and concentration, guilt and worthlessness, fatigue, movement, and suicidal ideation). For each item, respondents were asked to rate their experience in the past week on a 5-point scale (0 = “not at all or less than 1 day,” 1 = “1 or 2 days,” 2 = “3 or 4 days,” 3 = “5–7 days,” and 4 = “nearly every day for 2 weeks”). The scores were summed to a maximum total score of 60, with higher scores indicating more severe depression. The scale had a high internal consistency (Cronbach's α = 0.928) and a good construct validity in the general population of the United States.^38^ It also demonstrated high internal reliability among Chinese youth populations (Cronbach's α = 0.96).^39^

##### 2.3.2.2 Emotional and behavioral problems

The Strengths and Difficulties Questionnaire was adopted to measure emotional/behavioral problems.^40^ The scale has 25 items, with five items assessing each of the five subscales: perceived emotional problems, behavioral problems, hyperactivity, peer relationship problems, and prosocial behavior. Participants indicated responses on a 3-point Likert scale (0 = “not true,” 1 = ”somewhat true,” and 2 = “completely true”), with the scores for each subscale ranging from 0 to 10. This study focused on internalizing (combining emotional symptoms and peer relationship problems) and externalizing symptoms (including conduct problems and hyperactivity), with higher scores indicating more severe internalizing/externalizing problems.^41^ The Strengths and Difficulties Questionnaire demonstrated good internal consistency among Chinese children, and the Cronbach's α for internalizing and externalizing symptoms were 0.79 and 0.75, respectively.^42^

##### 2.3.2.3 Executive function

The Childhood Executive Functioning Inventory (CHEXI)^43^ and the Teenage Executive Functioning Inventory (TEXI)^44^ were used to assess children and adolescents' executive function, respectively. CHEXI is an instrument used by parents to evaluate children aged 4–12 years. It has 24 items and four dimensions such as working memory (e.g., "Has difficulty remembering lengthy instructions"), planning ability (e.g., "Has difficulty with tasks or activities that involve several steps"), regulation ability (e.g., "Has clear difficulties doing things he/she finds boring"), and inhibition ability (e.g., "Has difficulty holding back his/her activity despite being told to do so"). These dimensions are categorized into two factors: working memory (working memory and planning dimensions) and inhibition control (inhibition and regulation dimensions). TEXI is an instrument focusing on the executive function of adolescents between the ages of 13 and 19. The scale consists of 20 items, with 11 items measuring working memory(e.g., "I have difficulty remembering long instructions") and nine items measuring inhibition control (e.g., "I do things without first thinking about what could happen"). Both scales use a 5-point Likert scale, with scores ranging from 1 (definitely not true) to 5 (definitely true). Higher scores indicate greater working memory or inhibition control difficulties. CHEXI and TEXI demonstrated satisfactory internal consistency (Cronbach's α ranging from 0.80 to 0.88 across scales and subscales) in Chinese children^45^ and adolescents.^46^

#### 2.3.3 Confounders

The demographic information of the participants, such as sex, age, educational level of parents and annual household income, and body mass index (BMI), were included as confounders, which were reported to influence the 24-h movement behaviors and mental health of children and adolescents.^47^ The demographic information were self reported by the participants. Height and weight were recorded using a portable instrument. BMI was calculated as weight in kilograms divided by height in meters squared.

### 2.4 Data analysis

Compositional data analysis was utilized to generate the compositions of movement behavior for WDs and WEs by using R software (version 4.5.2). These time-use data exist in a constrained data space, where the sum of all parts can represent 1440 min. These adjusted data (including sleep, SB, LPA, and MVPA) were then transformed into two sets of three isometric log-ratio (ILR) coordinates, separately for WDs and WEs using the default Helmert orthonormal basis implemented in the "compositions" package. This transformation preserves the relative information of MB composition, allowing it to be used as real vectors in standard statistical models. The transformation formula is as follows: $ilri=\sqrt{\frac{i}{i+1}}In\left[\frac{x_{i+1}}{g\left(X_{1},...,x_{i}\right)}\right],i=$ 1, 2, …, D-1.

The ILRs were then used to identify the 24-h movement behavior profiles by using latent profile analysis on Mplus software (version 8.3). The covariances of ILRs were included in the latent profile model because ILRs are used to construct a coordinate system with a regular covariance matrix, and different ILR coordinate systems are just mutual rotations.^12^ The selection of the optimal latent profile model was guided by a combination of statistical fit indices and substantive criteria. The model fit was judged on the basis of the following three indices^48^^,^^49^: 1) the Akaike information criterion (AIC) and the Bayesian information criterion (BIC), which indicate the goodness of fit and simplicity, with lower values indicating better model fit); 2) the Lo–Mendell–Rubin-adjusted (LMRA) likelihood ratio test and bootstrap likelihood ratio test (BLRT), which assess the fitting difference between models with different numbers of profiles, where a significant result (P < 0.05) suggests that the model with k–1 profile provides a better fit than the k-profile solution; and 3) Entropy, which evaluates the classification precision of the model, with higher values indicating greater precision of model fit. Ultimately, the sample sizes (≥10% of the total sample size) and the interpretation of the profiles were considered in selecting the best classification model.^50^^,^^51^ Descriptive statistics for each profile were calculated using the individual's posterior probability (i.e., confidence) of assignment to a given profile as the analysis weight. Weighted averages, standard deviations, and weighted proportions were calculated. The chi-square test for categorical variables and the one-way ANOVA for continuous variables were used to assess differences in profiles.

To account for the bias of misclassification, the manual Bolck–Croon–Hagenaars (BCH) three-step approach was applied to examine differences in distal outcomes across latent profiles.^52^^,^^53^ Preliminary hierarchical models with school specified as a random effect indicated that different school-level did not significantly contribute to the variability of mental health outcomes. Therefore, all BCH models were adjusted for age, sex, BMI, parents' educational level, and family economic status. Wald tests were used to evaluate overall differences in mental health outcomes across latent profiles, and pairwise comparisons were conducted when the omnibus tests were statistically significant (p < 0.05). Effect sizes for statistically significant pairwise differences were calculated using Hedges’ g.^54^

## 3 Results

### 3.1 Descriptive characteristics of participants

The descriptive characteristics of primary and secondary school students are presented in Table 1. A total of 1219 participants, comprising 400 (32.8%) primary school students and 819 (67.2%) secondary school students, were involved in the study. The mean age was 13.1 years (SD = 3.0). The breakdown by gender was 602 (49.4%) boys and 617 (50.6%) girls. On average, the participants spent more time on WDs in SB by 31.1 min (678.2 vs. 647.1 min), in LPA by 11.3 min (200.8 vs. 189.5 min), and in MVPA by 10.0 min (48.7 vs. 38.7 min), but less time by 52.3 min in sleep (512.4 vs. 564.7 min) in comparison with WEs.

**Table 1:** Participant characteristics for whole sample, primary and secondary school students.

Variable	Total (N = 1219)	Primary school students (N = 400)	Secondary school students (N = 819)	*P*-value
*Demographic characteristics*				
Age, years	13.1 ± 3.0	9.4 ± 1.9	14.9 ± 1.4	< 0.001
Gender, girls, N (%)	617 (50.6)	191 (47.8)	426 (52.0)	0.181
Annual household income, N (%)				< 0.001
< 9000	241 (19.8)	111 (27.8)	130 (15.9)	
9000–30,000	459 (37.7)	148 (37)	311 (38)	
30,001–100,000	385 (31.6)	103 (25.8)	282 (34.4)	
> 100,000	134 (11)	38 (9.5)	96 (11.7)	
Father's education, N (%)				< 0.001
Primary school and below	76 (6.2)	24 (6.0)	52 (6.3)	
Junior high school	536 (44.0)	129 (32.2)	407 (49.7)	
High school	344 (28.2)	107 (26.8)	237 (28.9)	
Bachelor's degree	234 (19.2)	125 (31.2)	109 (13.3)	
Master's degree and above	29 (2.4)	15 (3.8)	14 (1.7)	
Mother's education, N (%)				< 0.001
Primary school and below	140 (11.5)	37 (9.3)	103 (12.6)	
Junior high school	508 (41.7)	125 (31.3)	383 (46.8)	
High school	337 (27.6)	106 (26.5)	231 (28.2)	
Bachelor's degree	214 (17.6)	125 (31.2)	89 (10.9)	
Master's degree and above	20 (1.6)	7 (1.8)	13 (1.6)	
BMI	20.0 ± 4.4	16.9 ± 3.0	21.6 ± 4.0	< 0.001
*Twenty-four-hour movement behavior*^a^				
Weekdays				
SB, min/day	678.2 ± 166.0	532.1 ± 91.2	749.6 ± 146.4	< 0.001
LPA, min/day	200.8 ± 76.4	275.9 ± 61.7	164.1 ± 52.2	< 0.001
MVPA, min/day	48.7 ± 22.7	62.0 ± 23.7	42.1 ± 19.2	< 0.001
Sleep time, min/day	512.4 ± 141.3	570.1 ± 95.0	484.2 ± 151.3	< 0.001
Weekend days				
SB, min/day	647.1 ± 198.5	492.9 ± 117.1	722.4 ± 186.3	< 0.001
LPA, min/day	189.5 ± 78.9	262.5 ± 76.7	153.8 ± 50.3	< 0.001
MVPA, min/day	38.7 ± 22.1	47.9 ± 29.7	34.1 ± 15.4	< 0.001
Sleep time, min/day	564.7 ± 192.8	636.6 ± 124.0	529.6 ± 210	< 0.001
*Depressive symptom*	16.6 ± 10.4	12.3 ± 9.1	18.7 ± 10.4	< 0.001
*Emotional and behavioral problems*				
Internalizing problems	6.1 ± 3.2	5.5 ± 3.2	6.3 ± 3.2	< 0.001
Externalizing problems	6.5 ± 3.0	6.2 ± 3.1	6.7 ± 3.0	0.002
*Executive function*				
Working memory	25.9 ± 8.0	30.0 ± 9.2	23.9 ± 6.4	< 0.001
Inhibition control	29.9 ± 8.2	29.1 ± 8.1	30.3 ± 8.3	0.017

Data are presented as mean ± SD unless otherwise indicated. N, number; SD, standard deviation; BMI, body mass index; SB, sedentary behavior; LPA, light-intensity physical activity; MVPA, moderate-to-vigorous physical activity.

a Compositional means for each movement behaviour.

### 3.2 Latent profiles of 24-h movement behavior during WDs and WEs

The model fit indices for 1–5 profiles are presented in Table 2. In primary school students, the AIC, BIC, and aBIC values decreased continuously as the number of profiles increased, favoring the five-profile model. Similarly, BLRT favored the five-profile model. However, the four- and five-profile models resulted in one profile that included three (0.08%) participants only. Subsequently, the AIC, BIC, aBIC, and BLRT statistics and the overall entropy statistics favored the three profiles, although LMR did not reach statistical significance. Therefore, the three-profile model was considered a solution for primary school students.

**Table 2:** Fit indicators for latent 1–5 profile models.

Profiles	AIC	BIC	aBIC	Entropy	*P*-value		Cases per class (n)
					LMR	BLRT	
*Primary school students (n = 400)*							
1	830.292	902.138	845.023				
2	723.003	822.790	743.463	0.697	0.0613	< 0.0001	309/91
3	**665.145**	**792.872**	**691.334**	**0.719**	**0.0795**	**< 0.0001**	**259/72/69**
4	610.866	766.533	642.784	0.813	0.2900	< 0.0001	206/153/38/3
5	583.050	766.657	620.696	0.734	0.2248	< 0.0001	178/104/81/34/3
*Ssecondary school students (n = 819)*							
1	3431.960	3516.705	3459.544				
2	3149.922	3267.624	3188.234	0.684	0.0005	< 0.0001	511/308
3	2970.581	3121.239	3019.620	0.713	0.0167	< 0.0001	419/229/171
4	**2876.067**	**3059.682**	**2935.833**	**0.739**	**0.0001**	**< 0.0001**	**389/211/116/103**
5	2823.941	3040.513	2894.435	0.747	0.1877	< 0.0001	294/193/124/117/91

Note. Boldface values indicate selected model.

AIC, Akaike information criterion; BIC, Bayesian information criterion; aBIC, sample-size-adjusted Bayesian information criterion; BLRT, bootstrapped likelihood ratio test; LMR, Lo–Mendell–Rubin-adjusted likelihood ratio test.

Similarly, the results showed that the AIC, BIC, aBIC, and BLRT statistics and the overall entropy statistics favored the five profiles in secondary school students. However, LMR suggested that the five-profile model did not provide a statistically significant improvement over the four-profile model. The four-profile solution was the optimal model for secondary school students according to these results and the parsimonious principle.

**Fig. 1:**
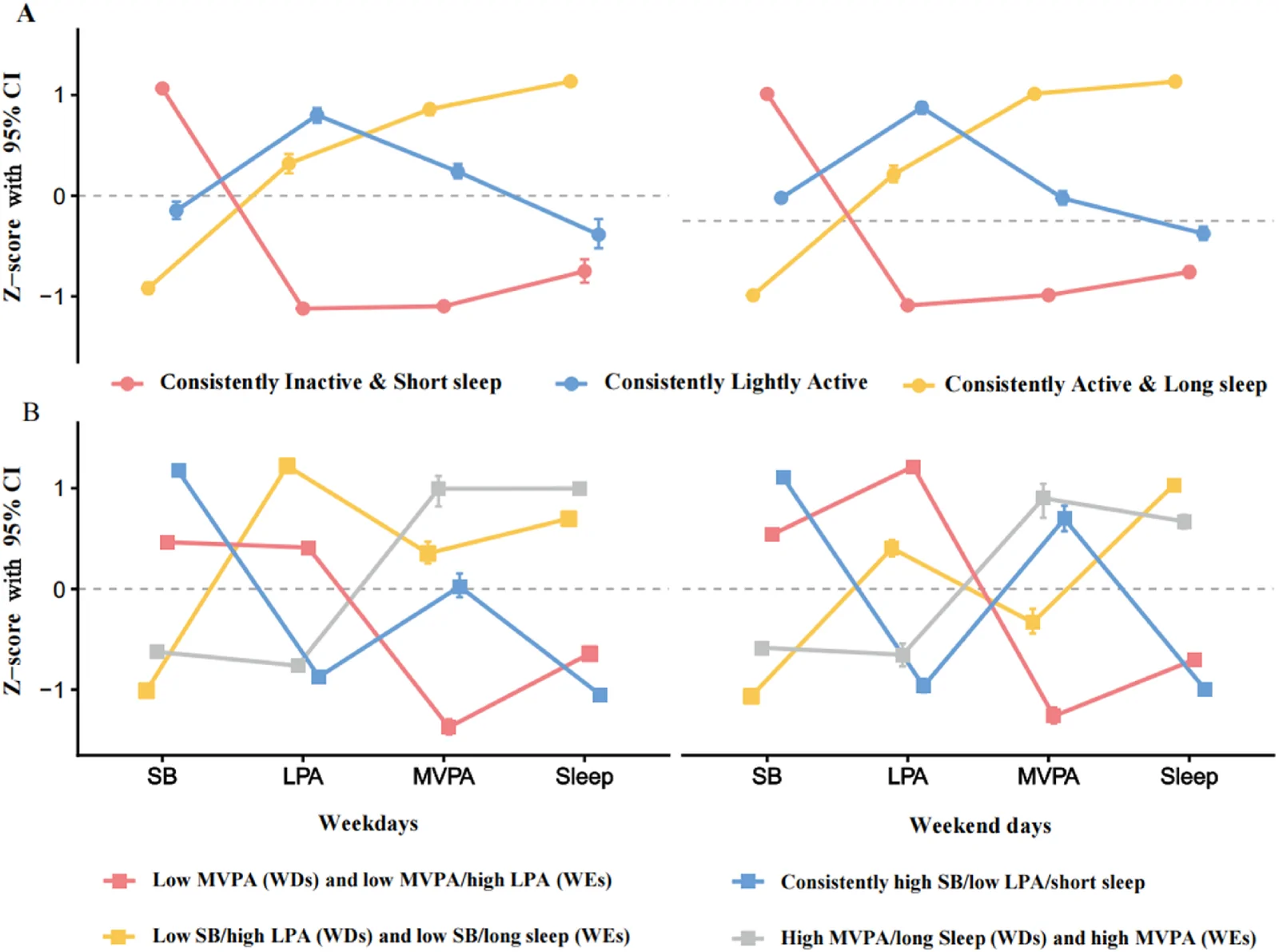
Z-score with 95% CI of 24-h movement behavior during WDs and WEs among distinct profiles of primary(A) and secondary(B) school students. Positive and negative values represent higher and lower compositional means than the whole population.

The profiles were labelled based on compositional mean of 24-h movement behavior (Table 3).Fig. 1 presents the mean z-scores of 24-h movement behavior compositions on WDs and WEs, calculated from the 24-h movement behavior compositions, illustrating the extent to which each profile was above or below the overall sample mean. Three profiles with consistent WD–WE patterns were identified among primary school students (Fig. 1A): “consistently low SB/high MVPA/long sleep” (72, 18%), “consistently high LPA” (259, 64.8%), and “consistently high SB/low PA/short sleep” (69, 17.3%). Among the identified latent profiles, the “consistently low SB/high MVPA/long sleep” group was characterized by spending the most time in MVPA and sleep and the least in SB. The “consistently high LPA” group showed the longest time in LPA. The “consistently high SB/low PA/short sleep” group engaged mostly in SB and the least in LPA, MVPA, and sleep. Given that the “consistently high SB/low PA/short sleep” group spent the most time in SB and had the least time in LPA, MVPA, and sleep, this group was considered the unhealthiest and selected as a reference for comparisons in primary school students.

**Table 3:** Participant characteristics for distinct profiles of primary and secondary school students.

Variable	Primary school students				Secondary school students				
	Consistently high SB/low PA/short sleep (N = 69)	Consistently high LPA (N = 259)	Consistently low SB/high MVPA/long sleep (N = 72)	*P*-value	Consistently high SB/low LPA/short sleep (N = 211)	Low MVPA (WDs) and low MVPA/high LPA (WEs) (N = 103)	Low SB/high LPA (WDs) and low SB/long sleep (WEs) (N = 116)	High MVPA/long sleep (WDs) and high MVPA (WEs) (N = 389)	*P*-value
*Demographic characteristics*									
Age, years	10.8 ± 1.5^a^	9.2 ± 1.8^b^	8.8 ± 1.8^b^	< 0.001	15.7 ± 1.1^a^	14.9 ± 1.4^b^	13.9 ± 1.1^c^	14.8 ± 1.4^b^	< 0.001
Gender, girls (%)	70.9^a^	49.4^b^	17.9^c^	< 0.001	67.1^a^	68.6^a^	33.3^b^	45.1^b^	< 0.001
Annual household income, %				0.004					0.010
< 9000	29.2^a^	29.7^a^	21.4^b^		12.8^a^	24.5^b^	19.6^b^	13.3^a^	
9000–30,000	41.2	39.6	21.6		32.8	37.2	37.8	41.5	
30,001–100,000	18.2	23.1	42.3		44.0	32.2	32.1	30.7	
> 100,000	11.4	7.6	14.7		10.5	6.0	10.5	14.5	
Father's education, %				<0.001					0.211
Primary school and below	13.1^a^	4.9^a^	4^b^		9.2	9.5	8.8	3.2	
Junior high school	35.9	35.5	15.9		48.1	51.2	48.7	48.9	
High school	20	28.7	24.7		27.7	24.8	25.8	32.6	
Bachelor's degree	26.5	28.8	46.3		14.4	12.2	14.0	13.3	
Master's degree and above	4.4	2.1	9.1		0.7	2.3	2.7	2.0	
Mother's education, %				0.022					0.138
Primary school and below	16^a^	9.3^a^	5.4^b^		16.2	15.1	13.2	10.1	
Junior high school	33.9	32.7	21		48.1	48.3	38.2	47.5	
High school	24.1	26.7	27.6		23.4	20.7	35.4	30.8	
Bachelor's degree	22.8	30.8	41.3		11.6	14.8	11.4	9.4	
Master's degree and above	3.3	0.5	4.7		0.7	1.1	1.8	2.2	
BMI, kg/m^2^	18.0 ± 3.5^a^	16.6 ± 2.9^b^	16.6 ± 2.7^b^	0.016	21.1 ± 3.3	22.0 ± 4.1	22.3 ± 4.7	21.5 ± 4.2	0.062
Twenty-four hour movement behavior^e^									
Weekdays									
SB, min/day	657.1 ± 60.8	531.8 ± 57.2	411.9 ± 53.9	< 0.001	914.9 ± 87.8	823.9 ± 88.5	625.8 ± 108.9	674.6 ± 105	< 0.001
LPA, min/day	218.3 ± 51.0	295.0 ± 55.7	267.4 ± 58.0	< 0.001	140.9 ± 31.8	199.6 ± 43	236.1 ± 55.5	145.2 ± 39.5	< 0.001
MVPA, min/day	38.7 ± 13.8	61.6 ± 18.4	85.8 ± 26.0	< 0.001	39.7 ± 13.9	31.3 ± 11.7	42.4 ± 21.7	46.4 ± 21.5	< 0.001
Sleep time, min/day	526.0 ± 75.9	551.6 ± 81.1	674.8 ± 86.6	< 0.001	344.6 ± 97.8	385.2 ± 100.9	535.6 ± 123.4	573.8 ± 126.7	< 0.001
Weekend days									
SB, min/day	629.0 ± 111.7	485.2 ± 88.8	394.7 ± 106.4	< 0.001	917.9 ± 111	829.1 ± 119.4	556.6 ± 120.6	633.8 ± 144	< 0.001
LPA, min/day	194.0 ± 47.1	284.0 ± 75.5	259.7 ± 71.3	< 0.001	137.4 ± 36.3	192.9 ± 50.7	172.2 ± 54.5	145.5 ± 47.3	< 0.001
MVPA, min/day	22.8 ± 12.2	50.8 ± 29.8	62.5 ± 30.1	< 0.001	35.5 ± 13.3	25.3 ± 10.8	30.4 ± 13.1	36.9 ± 17.1	< 0.001
Sleep time, min/day	594.2 ± 121.0	620.0 ± 114.8	723.1 ± 125.3	< 0.001	349.2 ± 128.8	392.7 ± 149.6	680.8 ± 163.8	623.7 ± 181.9	< 0.001
ILR units, mean									
Weekdays									
ILR1	0.15	−0.02	−0.31	-	0.67	0.53	−0.09	0.06	-
ILR2	−0.78	−0.52	−0.57	-	−1.16	−0.91	−1.06	−1.19	-
ILR3	−2.07	−1.74	−1.45	-	−2.04	−2.41	−2.27	−2.08	-
Weekend days									
ILR1	0.02	−0.18	−0.42	-	0.67	0.53	0.14	0.15	-
ILR2	−0.91	−0.57	−0.59	-	−1.14	−0.87	−0.78	−1.18	-
ILR3	−2.56	−1.95	−1.73	-	−1.94	−2.23	−2.06	−1.89	-

Data are presented as mean ± SD, unless otherwise indicated. BMI, body mass index; SB, sedentary behavior; LPA, light-intensity physical activity; MVPA, moderate-to-vigorous intensity physical activity; WDs, weekdays; WEs, weekend days. a,b,c,d Post-hoc test: the same letter indicates no significant difference. Any difference between two values carrying different letters is significant at 0.05.

e Compositional means for each movement behaviour.

The other four profiles were identified in secondary school students (Fig. 1B), such as “low MVPA (WDs) and low MVPA/high LPA (WEs)” (103, 12.6%), “consistently high SB/low LPA/short sleep” (211, 25.8%), “low SB/high LPA (WDs) and low SB/long sleep (WEs)” (116, 14.2%), and “high MVPA/long sleep (WDs) and high MVPA (WEs)” (389, 47.5%). Among the identified latent profiles, the “low MVPA (WDs) and low MVPA/high LPA (WEs)” group was engaged in the least MVPA time throughout the whole week and the most LPA time on WEs. The “consistently high SB/low LPA/short sleep” group had the most time in SB and the least time in LPA and sleep throughout the whole week. The “low SB/high LPA (WDs) and low SB/long sleep (WEs)” group was engaged in the least SB throughout the whole week, the most LPA on WDs, and the longest sleep on WEs. The “high MVPA/long sleep (WDs) and high MVPA (WEs)” group was characterized by the most time in MVPA throughout the whole week and the most time in sleep on WDs. Given that the “consistently high SB/low LPA/short sleep” group spent the most time in SB, the least time in LPA, and the least sleep throughout the whole week, this group was considered the unhealthiest and selected as a reference for comparisons in secondary school students.

### 3.3 Differences between groups

Table 3 displays the distribution of demographic information, BMI, and 24-h movement behavior on WDs and WEs for the distinct profiles of primary and secondary school students. Among primary school students, the “consistently high SB/low PA/short sleep” group was significantly older than the other two groups (10.8 ± 1.5 > 9.2 ± 1.8 and 8.8 ± 1.8, *P* < 0.01). The proportion of girls was the highest in the “consistently high SB/low PA/short sleep” group, followed by the “consistently high LPA” and “consistently high SB/low PA/short sleep” groups (70.9% > 49.4% > 17.9%, *P* < 0.01). The children in the “consistently low SB/high MVPA/long sleep” group were predominantly from higher socioeconomic status families, with a significantly higher proportion reporting an annual household income of over 100,000 RMB and parents with a bachelor's degree than the “consistently high LPA” and “consistently high SB/low PA/short sleep” groups (all *P* < 0.05). Moreover, the “consistently high SB/low PA/short sleep” group had the highest BMI (18.0 ± 3.5 > 16.6 ± 2.9 and 16.6 ± 2.7, *P* < 0.05) among the three groups.

Among secondary school students, the “consistently high SB/low LPA/short sleep” group was the oldest (15.7 ± 1.1), followed by the “low MVPA (WDs) and low MVPA/high LPA (WEs)” (14.9 ± 1.4), “high MVPA/long sleep (WDs) and high MVPA (WEs)” (14.8 ± 1.4), and “low SB/high LPA (WDs) and low SB/long sleep (WEs)” (13.9 ± 1.1) groups. The proportion of girls was significantly higher in the “consistently high SB/low LPA/short sleep” and “low MVPA (WDs) and low MVPA/high LPA (WEs)” groups than in the “high MVPA/long sleep (WDs) and high MVPA (WEs)” and “low SB/high LPA (WDs) and low SB/long sleep (WEs)” groups (67.1% and 68.6% > 45.1% and 33.3%; *P* < 0.05). The adolescents in the “low MVPA (WDs) and low MVPA/high LPA (WEs)” and “low SB/high LPA (WDs) and low SB/long sleep (WEs)” groups were predominantly from low-income families, with a significantly higher proportion reporting an annual household income of less 9000 RMB than the “high MVPA/long sleep (WDs) and high MVPA (WEs)” and “consistently high SB/low LPA/short sleep” groups (24.5% and 19.6% > 13.3% and 12.8%; *P* < 0.05).

### 3.4 Differences in mental health outcomes across 24-h movement behavior profiles

Table 4 shows the differences in mental health outcomes across the distinct profiles in primary and secondary school students, adjusted for confounders. Among primary school students, the results of the overall χ2 test indicated the overall differences in depression, internalized problems, working memory, and inhibitory control (all *P* < 0.05). Pairwise comparisons revealed that the “consistently low SB/high MVPA/long sleep” group had a significantly lower depression level (M = 13.6, SE = 2.1, *P* = 0.023), fewer internalized problems (M = 6.1, SE = 0.8, *P* = 0.029), and better working memory (M = 33.8, SE = 2.5, *P* = 0.009) and inhibition control (M = 27.4, SE = 2.3, *P* = 0.031) than the “consistently high SB/low PA/short sleep” group. Similarly, the “consistently high LPA” group had a significantly lower depression level (M = 14.5, SE = 1.9, *P* = 0.036), fewer internalized problems (M = 6.2, SE = 0.7, *P* = 0.012), and better working memory (M = 34.3, SE = 2.1, *P* = 0.002) and inhibition control (M = 27.8, SE = 1.8, *P* = 0.01) than the “consistently high SB/low PA/short sleep” group. No significant difference in the association with mental health of the “consistently low SB/high PA/long sleep” and “consistently high LPA” groups. No significant associations of 24-h movement behavior profiles with externalizing problems were observed.

**Table 4:** Mean and pairwise comparisons of mental health outcomes across distinct 24-h movement behavior profiles in primary and secondary school students.

Profile	Depressive symptom	Internalizing problems	Externalizing problems	Working memory	Inhibition control
**Primary school students**					
1- Consistently high SB/low PA/short sleep^a^	18.7 ± 2.7	7.9 ± 1.0	7.3 ± 1.0	39.9 ± 2.8	31.8 ± 2.4
2- Consistently high LPA	14.5 ± 1.9*	6.2 ± 0.7*	6.4 ± 0.7	34.3 ± 2.1*	27.8 ± 1.8*
3- Consistently low SB/high MVPA/long sleep	13.6 ± 2.1*	6.1 ± 0.8*	6.4 ± 0.9	33.8 ± 2.5*	27.4 ± 2.3*
Hedges' g	P1 vs. P2: 0.430 P1 vs. P3: 0.522	P1 vs. P2: 0.503 P1 vs. P3: 0.543	-	P1 vs. P2: 0.629 P1 vs. P3: 0.608	P1 vs. P2: 0.517 P1 vs. P3: 0.515
**Secondary school students**					
1-Consistently high SB/low LPA/short sleep^a^	16.0 ± 2.1	6.3 ± 0.6	6.7 ± 0.6	21.8 ± 1.2	27.0 ± 1.6
2-Low MVPA (WDs) and low MVPA/high LPA (WEs)	13.2 ± 2.0	5.5 ± 0.6	6.6 ± 0.6	20.7 ± 1.2	25.3 ± 1.7
3-Low SB/high LPA (WDs) and low SB/long sleep (WEs)	15.3 ± 2.1	6.0 ± 0.7	7.2 ± 0.7	23.1 ± 1.4	26.2 ± 1.8
4-High MVPA/long sleep (WDs) and high MVPA (WEs)	13.3 ± 1.8*	5.2 ± 0.6*	6.6 ± 0.6	21.0 ± 1.2	25.6 ± 1.5
Hedges' g	P1 vs. P4: 0.254	P1 vs. P4: 0.340	-	-	-

Data are presented as mean ± SE. SB, sedentary behavior; LPA, light-intensity physical activity; MVPA, moderate-to-vigorous intensity physical activity; WDs, weekdays; WEs, weekend days. Means and standard errors (SE) are adjusted for classification uncertainty using the manual BCH method. ^a^ reference group. * significant differences at *p* < 0.05 level in a BCH weighted mean difference test with Holm's Bonferroni-adjusted *p*-value.

In secondary school students, the results of the overall χ2 test indicated the overall differences in depression and internalized problems (all *P* < 0.05). Pairwise comparisons revealed that when using “consistently high SB/low LPA/short sleep” as the reference group, the “high MVPA/long sleep (WDs) and high MVPA (WEs)” group had significantly lower depression levels (M = 13.3, SE = 1.8, *P* = 0.029) and fewer internalized problems (M = 5.2, SE = 0.6, *P* = 0.004). No significant differences in depression and internalized problems existed among the “low MVPA (WDs) and low MVPA/high LPA (WEs),” “low SB/High LPA (WDs) and low SB/long sleep (WEs),” and “consistently high SB/low LPA/short sleep” groups. Moreover, no significant associations were observed between 24-h movement behavior profiles and working memory, inhibition control, or externalizing problems.

## 4 Discussion

In this study, three and four distinct 24-h movement behavior profiles were identified in primary and secondary school students, respectively. Among primary school students, the “consistently low SB/high MVPA/long sleep” and “consistently high LPA” groups reported better levels of mental health than the “consistently high SB/low PA/short sleep” group. In terms of secondary school students, the “high MVPA/long sleep (WDs) and high MVPA (WEs)” group had lower depression levels and fewer internalized problems than the “consistently high SB/low LPA/short sleep” group.

### 4.1 Twenty-four-hour movement behavior profiles in primary and secondary school students

The three profiles identified in this study revealed consistency in the 24-h movement behavior patterns between WDs and WEs in primary school students. However, divergence existed in the four profiles between WDs and WEs in secondary school students. Further comparison of the movement behavior components of adolescents between WDs and WEs revealed that the differences mainly existed in LPA and sleep duration. This finding may be attributed to three reasons. First, a group of Chinese local studies has shown that the sleep duration of secondary school students in China is insufficient on WDs due to the high academic burden (i.e., studying late into the night) and daily school schedule (i.e., waking up early to go to school in the morning).^55^, ^56^, ^57^ The descriptive results of the current study confirmed that the average sleep duration of participants on WDs (484 min) did not reach the limit of the sleep duration recommendation (sleep durations of 8–10 h per night) from the 24-h movement behavior guideline in Canada.^58^ As a result, secondary school students may “compensate” for insufficient sleep with an extended sleep duration and later wake times on WEs due to a free schedule.^59^ Second, no compulsory classes such as Chinese, mathematics, English, and history, where secondary school students have to sit and listen to teachers, are held on WEs, which may increase adolescents' LPA (e.g., walking and playing) as a substitute for sitting, leading to the inconsistency in LPA between WDs and WEs. Third, secondary school students may have more autonomy (Tashjian et al., 2019), whereas primary school students’ daily routines are more strictly regulated by parental oversight (Rodriguez et al., 2009), which helps maintain 24-h movement behavior profile consistency across the week.

Additionally, 64.8% of primary school students in this study fell into the “consistently high LPA” profile, and 47.5% of secondary school students belonged to the “high MVPA/long sleep (WDs) and high MVPA (WEs)” profile, each representing the highest percentage within their respective age group. Most of the primary and secondary school students were classified into profiles characterized by relatively favorable movement behavior profiles with high MVPA, LPA, or long sleep. These findings align with those of previous research identifying the “active” profile as a predominant typology among children and adolescents (^12^; Padmapriya et al., 2021). However, the average MVPA duration across the week and WD sleep duration among secondary school students per day (i.e., 41.1 min of MVPA on WDs and 34.1 min on WEs and 484.2 min of sleep on WDs) remain below the recommended 60 min/day MVPA and 8–10 h/night of sleep outlined in the 24-Hour Movement Guidelines for Children and Youth.^58^ Therefore, interventions are needed to improve the 24-h movement behavior of secondary school students.

### 4.2 Association between 24-h movement behavior profiles and mental health in primary school students

Among the three movement behavior profiles of primary school students, “consistently low SB/high MVPA/long sleep” exhibited the most favorable mental health outcomes. This result aligns with expectations, given that this group was characterized by the most total MVPA and sleep time and the least total time in SB. This finding is also consistent with most evidence indicating that increased MVPA and sleep and lessened SB are strongly associated with reduced risks of depression and anxiety, as well as superior executive function.^60^, ^61^, ^62^

The “consistently high LPA” profile with the longest LPA duration showed significant benefits for mental health compared with the reference (i.e., “consistently high SB/low PA/short sleep”). This finding is consistent with those of previous studies reporting that LPA enhances executive function^63^ and mood^64^ in children. The central nervous systems of primary school students are highly plastic.^65^ Research indicated that low-intensity PA may benefit mental health by regulating brain systems involved in emotional control. Specifically, low-intensity activity could promote the activity of serotonin-related pathways, which are associated with improved mood and reduced anxiety, without excessively activating stress-related neural responses.^6^ Furthermore, LPA may increase cerebral blood flow in the prefrontal cortex, a brain region involved in attention, self-regulation, and emotional control, thus enhancing executive function and psychological mood in children.^5^

Notably, the identified 24-h movement behavior profiles showed no significant differences with externalizing problems, which refer to overt disruptive behaviors such as fighting, hyperactivity, truancy, and delinquency.^66^ Previous studies examined the association of meeting the Canadian 24-h movement guidelines for children and youth with externalizing behaviors among U.S. youth aged 9–11 years and achieved different findings.^67^^,^^68^ Sampasa-Kanyinga et al.^68^ found that meeting the 24-h movement guidelines was associated with a decreased risk of externalizing behaviors, particularly screen time and sleep duration recommendations. Fung et al.^67^ tracked 9273 U S. children aged 9–11 years for 2 years and found that adherence to recommended screen time and sleep duration is associated with diminished externalizing problems. The discrepancy may be attributed to the difference in sample age (9–11 years vs. 6–12 years), country (USA vs. China), and the measurement tools (accelerometer vs. questionnaire). Moreover, accelerometers cannot distinguish dysregulated movement (e.g., hyperactivity commonly observed in children with externalizing behaviors) from purposeful, health-enhancing physical activity. Consequently, accelerometer-derived movement profiles may reflect both health-promoting activity and symptoms of hyperactivity, potentially masking the association between movement behavior profiles and externalizing problems. Future studies should combine objective movement monitoring with behavioral assessments and adopt longitudinal cross-cultural approaches to further clarify the relationship between 24-h movement behavior profiles and externalizing problems.

In summary, primary school students can enhance their psychological well-being and cognitive functions by optimizing their 24-h movement behavior, specifically by increasing MVPA, decreasing SB, and ensuring adequate sleep duration. Additionally, maintaining high levels of LPA may exert a remarkable influence on primary school students’ mental health.

### 4.3 Association between 24-h movement behavior profiles and mental health in secondary school students

The results on secondary school students revealed that only the “high MVPA/long sleep (WDs) and high MVPA (WEs)” group exhibited significantly lower depression levels and fewer internalized problems than the “consistently high SB/low LPA/short sleep” group, consistent with previous studies reporting that the healthiest profile was associated with enhanced mental health.^15^^,^^16^ No significant differences existed among other groups. These findings emphasized the importance of MVPA and sleep time for mental health in secondary school students, in contrast with the results on primary school students, in which high MVPA or LPA was associated with enhanced mental health. From a neurobiological perspective, PA induces cellular and molecular changes that enhance neuroplasticity^69^ and improve the structure and function of several brain regions, such as the hippocampus,^70^ thereby improving mental health.^71^ Childhood is a highly sensitive period for neural development, characterized by cortical surface area and thickness typically peaking in late childhood.^72^ This developmental trajectory suggests a lower neural response threshold to external stimuli during childhood, such that even mild stimulation from LPA is sufficient to trigger neuroplastic adaptations. Conversely, as secondary school students undergo synaptic pruning and structural consolidation, the brain becomes more structurally stable.^73^ Consequently, secondary school students may require the greater physiological stress induced by MVPA to effectively drive similar neuro-structural optimization. Moreover, secondary school students often experience insufficient sleep on weekdays due to increasing academic demands and early-morning schedules and compensate for accumulated sleep debt by extending sleep duration on weekends.^74^ This discrepancy between weekday and weekend sleep patterns may result in social jetlag,^75^ which has been associated with mood disorders and cognitive problems.^76^, ^77^, ^78^ Therefore, sleep-related movement behaviors may be particularly relevant for mental health among secondary school students.

Notably, no significant differences were found in working memory, inhibitory control, or externalizing problems among the four groups of secondary school students. Previous studies have shown that prolonged engagement in academic SB, such as reading, writing, and homework, is favorably associated with cognitive development in adolescents.^79^^,^^80^ Meanwhile, increases in MVPA, LPA, and sufficient sleep time have been reported to be important factors promoting the cognitive development of secondary school students.^81^, ^82^, ^83^ Therefore, no significant differences in working memory and inhibitory control can be detected among the four groups with increased MVPA, LPA, SB, or sleep time. Moreover, previous studies indicated that PA, sleep, and screen time often exert an indirect influence on externalizing symptoms through mediating variables such as sleep, self-esteem, emotional regulation, and executive function.^84^, ^85^, ^86^, ^87^This finding may explain why no direct association was found between 24-h movement behavior profiles and externalizing symptoms in the present study. Future research could further examine the mediating effects to elucidate the specific pathways through which combined 24-h movement behavior influences externalizing symptoms.

In conclusion, strategies to promote mental health among secondary school students should prioritize ensuring adequate sleep and encouraging regular participation in MVPA.

### 4.4 Strengths and limitations

This study is the first to employ a data-driven and person-centered method to derive 24-h movement behavior profiles and examine their associations with mental health in Chinese primary and secondary school students. Additionally, the application of compositional data analysis provides a holistic perspective on movement behavior, facilitating the identification of profiles that encapsulate the integrated activity patterns. However, several limitations should be considered. First, the cross-sectional design precludes causality between 24-h movement behavior profiles and mental health. Future longitudinal studies are necessary to verify the relationship among these variables. Second, the participants were from six schools and two cities in China, thus restricting the generalizability of the results. A larger sample of multiple schools and more samples should be targeted in future research. Third, mental health outcomes were assessed using parent reports for primary school students and self-reports for secondary school students, which may introduce informant-related bias, including social desirability and the influence of parents’ psychological states.^88^ Therefore, differences in reporting sources may have affected the comparability of the findings between the two age groups, and the observed age-group differences should be interpreted with caution. Additionally, this study did not consider the distinctions between different accelerometer-derived metrics (e.g., activity counts, average acceleration, and milligravity) in 24-h movement behavior because these metrics may capture distinct physiological and behavioral constructs. Future studies should examine these metric differences because they may form typologies with distinct mental health outcomes.

## 5 Conclusion

This study identified three distinct 24-h movement behavior profiles in primary school students and four profiles in secondary school students. In primary school students, the “consistently low SB/high MVPA/long sleep” and “consistently high LPA” groups had significantly lower depression levels, fewer internalized problems, better working memory, and better inhibition control lower levels than the “consistently high SB/low PA/short sleep” group. In secondary school students, the “high MVPA/long sleep (WDs) and high MVPA (WEs)” group had significantly lower depression levels and fewer internalized problems than the “consistently high SB/low LPA/short sleep” group. Optimizing 24-h movement behavioral composition is critical for promoting the mental health of primary and secondary school students. Mental health promotion strategies should aim to optimize 24-h movement behaviors (i.e., increasing MVPA, decreasing SB, and ensuring adequate sleep duration) or maintain high levels of LPA for primary school students. For secondary school students, they should focus on prioritizing ensuring adequate sleep and encouraging regular participation in MVPA.

## Declaration of conflicting interests

Author Li-juan Wang is a member of the Editorial Board of Journal of Exercise Science and Fitness. Li-juan Wang was not involved in the journal's peer review process of, or decisions related to, this manuscript.
